# Specific effects of EEG based neurofeedback training on memory functions in post-stroke victims

**DOI:** 10.1186/s12984-015-0105-6

**Published:** 2015-12-01

**Authors:** Silvia Erika Kober, Daniela Schweiger, Matthias Witte, Johanna Louise Reichert, Peter Grieshofer, Christa Neuper, Guilherme Wood

**Affiliations:** Department of Psychology, University of Graz, Universitaetsplatz 2/III, Graz, 8010 Austria; BioTechMed-Graz, Graz, Austria; Klinik Judendorf-Strassengel, Gratwein-Strassengel, Austria; Laboratory of Brain-Computer Interfaces, Institute for Knowledge Discovery, Graz University of Technology, Graz, Austria

**Keywords:** Cognitive rehabilitation, EEG, Neurofeedback, Memory, Stroke recovery

## Abstract

**Background:**

Using EEG based neurofeedback (NF), the activity of the brain is modulated directly and, therefore, the cortical substrates of cognitive functions themselves. In the present study, we investigated the ability of stroke patients to control their own brain activity via NF and evaluated specific effects of different NF protocols on cognition, in particular recovery of memory.

**Methods:**

*N* = 17 stroke patients received up to ten sessions of either SMR (*N* = 11, 12–15 Hz) or Upper Alpha (*N* = 6, e.g. 10–12 Hz) NF training. *N* = 7 stroke patients received treatment as usual as control condition. Furthermore, *N* = 40 healthy controls performed NF training as well. To evaluate the NF training outcome, a test battery assessing different cognitive functions was performed before and after NF training.

**Results:**

About 70 % of both patients and controls achieved distinct gains in NF performance leading to improvements in verbal short- and long-term memory, independent of the used NF protocol. The SMR patient group showed specific improvements in visuo-spatial short-term memory performance, whereas the Upper Alpha patient group specifically improved their working memory performance. NF training effects were even stronger than effects of traditional cognitive training methods in stroke patients. NF training showed no effects on other cognitive functions than memory.

**Conclusions:**

Post-stroke victims with memory deficits could benefit from NF training as much as healthy controls. The used NF training protocols (SMR, Upper Alpha) had specific as well as unspecific effects on memory. Hence, NF might offer an effective cognitive rehabilitation tool improving memory deficits of stroke survivors.

## Background

Approximately two-thirds of stroke patients experience cognitive impairment following stroke including failures in executive functions, memory, language, visuo-spatial abilities, or global cognitive functioning [1]. Traditional cognitive rehabilitation methods have not proven fruitful or they have not been evaluated sufficiently yet [2–4]. A recent review by Elliott and Parente (2014) indicated that on the one hand traditional memory rehabilitation is an effective therapeutic intervention after stroke, especially to improve working memory performance, but on the other hand significant memory improvement also occurred spontaneously over time [5]. Some major drawbacks of traditional cognitive rehabilitation are the employment of similar tasks for training and evaluation of outcomes, the requirement of overt responses from the patients, its dependence on relatively complex verbal instructions, and the requirement of a lot of cognitive effort. The aim of the present study was to evaluate a new rehabilitation strategy suitable to overcome the usual pitfalls of traditional cognitive rehabilitation. An adaptive human-computer interface architecture for improving cognition, in particular memory, was evaluated. This setup modulated electrical brain activity using Electroencephalogram (EEG) based neurofeedback (NF) as a cognitive rehabilitation tool for stroke patients.

Using EEG based NF, the electrical activity of the brain is modulated directly and, therefore, the cortical substrates of cognitive functions themselves. This direct access to neural activity by means of NF may alter or accelerate functional reorganization in the brain following stroke, indicating the great potential value of NF in cognitive rehabilitation. Hence, NF might speed up functional recovery or even enable functional recovery that otherwise would not have occurred [6]. When healthy participants successfully modulate their own brain activity, e.g. increasing voluntarily specific EEG frequency bands, improvements in cognition and behavior usually follow [7, 8].

In the present study, we evaluated the effects of NF training on memory in stroke patients. We used two NF training protocols with beneficial effects on memory in healthy people: SMR (sensorimotor rhythm, 12–15 Hz) and UA (Upper Alpha, e.g. 10–12 Hz) based NF. In prior studies, healthy participants, who were trained to increase their SMR power, showed improvements in declarative memory performance [7–12], referring to memories which can be consciously recalled such as facts and knowledge [13]. Generally, SMR is largest over central scalp regions over the sensorimotor cortex, it is generated in a thalamo-cortical network, emerges when one is motionless yet remains attentive, and is suppressed by movement [14–16]. This EEG rhythm is associated with “internal inhibition”, since there is evidence that during SMR activity the conduction of somatosensory information to the cortex is attenuated or inhibited [15]. This inhibition of somatosensory information flow to the cortex during increased SMR activity is associated with improved cognitive performance. Sterman (1996) proposed that motor activity may interfere with perceptual and integrative components of information processing, since motor activity can disengage visual processing areas of the cortex. Such sensorimotor interference with visual processing may hamper cognitive performance [15, 17]. In this context, voluntary control of sensorimotor excitability by means of SMR based NF training may facilitate cognitive processing by decreasing sensorimotor interference and maintaining perceptual and memory functions at the same time [15]. In line with this assumption, Kober et al. (2015) could show that SMR based NF training leads to reduced sensorimotor interference and can thereby promote cognitive processing in healthy people [9].

Increasing UA activity by means of NF training also causes memory improvements, especially improvements in working memory (WM) and short-term memory performance [18–24]. Alpha oscillations are generally most pronounced over parieto-occipital areas [25]. It is assumed that alpha activity inhibits processes unnecessary for or conflicting to the task being performed, thus facilitating attention and memory by actively suppressing distracting stimuli [26]. Klimesch (1999) proposed to split up the alpha frequency range (e.g. 8–12 Hz) in a lower (e.g. 8–10 Hz) and upper (e.g. 10–12 Hz) alpha band, since the upper and lower alpha frequency range were linked to different cognitive processes. While lower alpha activity is associated with attentional processes that are relatively task- and stimulus-non-specific, upper alpha activity is specifically related to memory performance. In particular, search and retrieval processes are reflected by upper alpha oscillations [25].

Several NF studies in healthy participants reported on a link between the ability to gain control over EEG signals and cognitive benefits [8]. Similar results were observed in patients with traumatic brain injury (TBI) [2, 27] and stroke [28–31]. Single-case studies in stroke patients found positive but unspecific NF training effects on cognitive functions [28–32]. However, the generalizability of these prior findings is limited due to the incomplete description of training-specific EEG signal changes as well as the absence of control groups. A study by Hofer et al. (2014) is one of the first NF training studies investigating the effects of SMR and Theta/Beta quotient (4-8/13-21 Hz, T/B NF training) based NF training on cognitive functions in stroke patients and healthy controls [33]. The authors could demonstrate that stroke patients with memory impairments showed specific performance improvements in declarative memory tasks after SMR NF training, while stroke patients with deficits in attention and inhibition showed specific improvements in inhibitory control and cognitive flexibility after repeated T/B NF training.

In summary, a few prior studies indicated that NF might be a promising new treatment for cognitive rehabilitation after stroke [6]. The present study addressed the following open questions: First, are stroke patients comparable to controls regarding the ability to modulate their EEG signal using NF? Second, is the impact of NF specific regarding cognitive functions such as memory in stroke patients, or are NF effects more general (e.g., global cognitive functioning) [6]? We used two NF training protocols (SMR, UA), which should have beneficial effects on different memory functions, and investigated training effects on attention, inhibition, cognitive flexibility, short- and long-term memory and WM in stroke patients. Based on previous investigations, we hypothesized that stroke patients should be able to modulate their EEG activity voluntarily by means of NF training [28–31, 33]. Furthermore, we expected that SMR and UA based NF protocols should have specific effects on memory in both stroke patients and controls [8]. Based on the literature, SMR based NF training should have specific effects on declarative memory performance, whereas UA based NF training should specifically affect working memory performance. Furthermore, a comparable control group of stroke patients was employed who received treatment as usual. Hence, we could directly compare the effects of NF training with the effects of traditional cognitive rehabilitation methods on cognitive functions in stroke patients. As the electrophysiological balance is disturbed after brain lesion [2], any intervention might even accentuate this disturbance and thus may result in negative impact on cognition. Therefore, particular attention was given to the inspection of deleterious effects of NF on EEG and cognition as well.

## Methods

### Participants

We recruited 24 stroke patients with first-time stroke for this study. Table 1 summarizes patient specific data. As this study was a proof-of-principle study, we included stroke patients with any site of brain lesion and motor deficit and with a time laps from the event of at least 4 weeks. With regard to the drug therapies administered, patients treated with drugs that interfere with the vigilance state were not included. Furthermore, all participants had normal or corrected-to-normal vision and hearing. Patients with visual hemi-neglect, dementia (MMSE < 24, [34]), psychiatric disorders such as depression or anxiety, other concomitant neurological disorders (e.g. Parkinson disease; visual-reflex epilepsy), aphasia, or insufficiently motivation and cooperation were excluded from the study. All participants gave written informed consent to participate. We have also obtained consent from the participants to publish and to report individual patient data. The study was approved by the local ethics committee of the University of Graz (reference number GZ. 39/22/63 ex 2011/12 and GZ. 39/21/63 ex 2011/12) and was in line with the code of ethics of the World Medical Association, Declaration of Helsinki.

**Table 1:** Patient description

Code	Number of NF training sessions	Sex	Age	Handedness	ICD-10 diagnosis	Lesion location	Time since onset (days)	MMSE	NF performance
SMR NF training									
1	8	M	65	Lt	I63.5	Rt posterior	41	29	+
2	7	M	64	Rt	I63.5	Lt internal carotid artery	98	24	+
3	7	M	51	Rt	I61.9	Lt basal ganglia	2869	29	-
4	9	M	64	Rt	I63.9	Lt thalamus, arteria cerebri posterior (occipital-medial)	30	29	-
5	10	M	74	Rt	I63.1	Bt cerebellum (rt), hippocampus (bt), mesencephalon (rt), occipital lobe (lt), splenium (bt)	136	29	+
6	10	M	62	Rt	I61.9	Lt arteria cerebri media, occipital-parietal	1783	26	+
7	10	F	52	Rt	I63.9	Rt basilar artery, pons- mesencephalon	693	29	+
8	9	F	37	Rt	I60.2	Rt arteria communicans posterior, temporal	87	28	+
9	10	M	65	Rt	I63.5	Lt arteria cerebri posterior	78	28	+
10	10	M	62	Rt	I63.5	Lt arteria cerebri media	247	29	-
11	10	M	50	Rt	I64	Bt basal ganglia, corpus collosum (truncus, genu), inferior temporal	2714	29	+
Upper Alpha NF training									
12	10	M	72	Rt	I61.9, I60.9	Bt arteria cerebri media, occipital-parietal, frontal	808	30	+
13	6	M	73	Rt	I63.3	Lt arteria cerebri media	2111	29	+
14	8	F	82	Rt	I63.9	Lt pons	104	28	-
15	10	F	53	Rt	I63.5	Rt arteria cerebri media	930	30	-
16	10	M	76	Rt	I63.3	Rt arteria cerebri media	133	24	+
17	5	M	71	Rt	I63.9	Rt arteria cerebri media	362	29	+
Treatment as usual									
18		M	75	Rt	I63.9	Rt arteria cerebri media	87	27	
19		M	57	Rt	I63.5	Lt arteria cerebri media	93	27	
20		M	78	Rt	I63.9	Lt posterior occipital, cerebellum	88	28	
21		M	64	Rt	I63.3	Rt arteria cerebri media	61	24	
22		M	49	Rt	I63.5	Lt capsula interna	32	28	
23		W	61	Rt	I60.9	Lt arteria communicans interior	138	29	
24		M	71	Rt	I63.8	Rt cerebellum	43	29	

*Bt* bilateral, *F* female, *Lt* left, *M* male, *MMSE* mini-mental state examination, and Rt right. *NF* neurofeedback performance: “+” indicates that the patient was able to linearly increase the trained frequency band, “-“ indicates that the patient was not able to linearly increase the trained frequency band

Stroke patients showed deficits (T-scores < 40) in tests assessing verbal (CVLT: California Verbal Learning Test, all parameters, [35]; VVM2: Visual and Verbal Memory Test, subscale “construction”, [36]) and visuo-spatial (VVM2: subscale “city map”, [36]) short- and long-term memory performance during the pre-assessment (Fig. 3). In line with previous NF training studies [33, 37], stroke patients were assigned to the NF protocols depending on their most prominent cognitive deficits as assessed before the start of the NF training. *N* = 11 (9 men, 2 women; mean age 58.72 years, *SE* = 3.08; age range 37–74 years) stroke patients with memory deficits, especially with deficits in long-term memory performance, performed SMR (12–15 Hz) based NF training. *N* = 6 (4 men, 2 women; mean age 71.17 years, *SE* = 3.98; age range 53–82 years) stroke patients with memory deficits, especially with deficits in their WM performance, participated in an UA NF training (training frequency: 2 Hz above the individual Alpha frequency, [25]). Furthermore, *N* = 7 (6 men, 1 women; mean age 65.00 years, *SE* = 3.91; age range 49–78 years) stroke patients with memory deficits received traditional cognitive training during their stationary stay in the rehabilitation clinic Judendorf-Strassengel, Austria. This group forms the treatment as usual (TAU) group. Treatment as usual was comparable to NF training in terms of training frequency and duration. Note that it was not possible to perform all neuropsychological tests with the TAU group during the pre- and post-assessment, since not all tests were available at the clinic and because of economic reasons. The cognitive profiles of patients receiving either SMR NF training, UA NF training or TAU are illustrated in Fig. 3.

Additionally, a neurologically healthy control group (CG) (*N* = 40; 17 men, 23 women; mean age 59.63 years, *SE* = 1.41; age range 41–73 years) was recruited. *N* = 16 (9 men, 7 women; mean age 55.13 years, *SE* = 2.65; age range 41–70 years) controls performed SMR NF training and *N* = 24 (8 men, 16 women; mean age 62.63 years, *SE* = 1.25; age range 50–73 years) controls received UA based NF training. The healthy CG showed no deficits in any test parameter (Fig. 3).

**Fig. 1:**
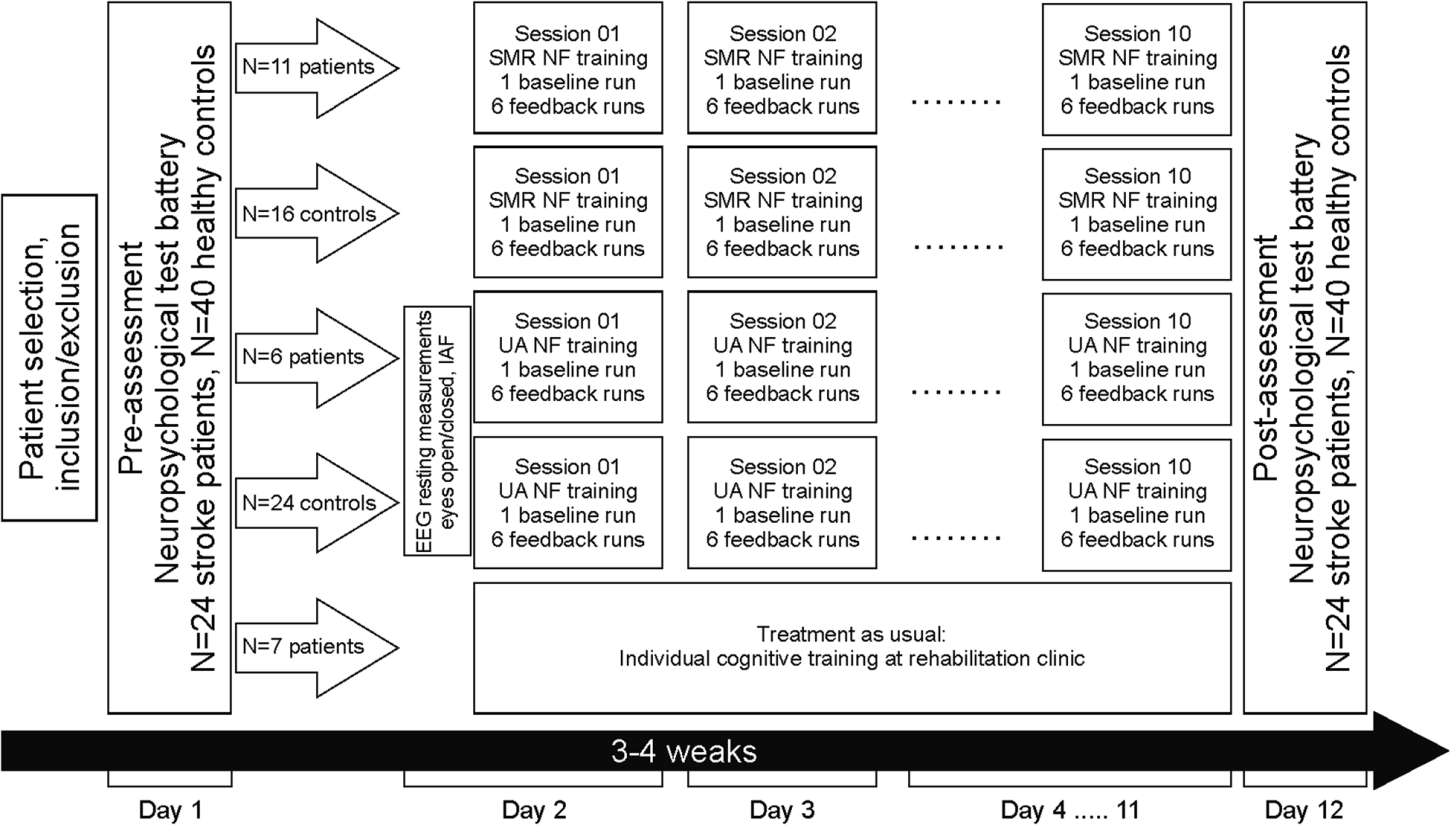
Design of the whole study, demonstrating the procedure for each group

Fig. 1 illustrates the design of the whole study in more detail.

### Neuropsychological assessment of cognitive functions

In pre- and post-assessment (before and after NF/cognitive training) participants performed standardized neuropsychological tests to assess attention, divided attention, inhibition, cognitive flexibility, declarative memory (long-term memory), short-term memory, and WM. The pre- and post-assessment was performed a few days before and after training, respectively. In Table 2 the list of neuropsychological tests assessing different cognitive functions can be found.

**Table 2:** List of neuropsychological tests assessing cognitive functions performed during the pre- and post-assessment

Cognitive function		Neuropsychological test	Analyzed test parameters
Attention	Alertness	Subtest Alertness of the TAP test battery [60]	RT without sound, RT with sound
	Divided Attention	Subtest Divided Attention of the TAP test battery [60]	RT auditory, RT visual, total errors, total omissions
Executive functions	Cognitive flexibility	Subtest Flexibility of the TAP test battery [60]	RT, errors, total performance index
	Inhibitory control	Subtest Go/NoGo of the TAP test battery [60]	RT, total errors, total omissions
Memory	Long-term memory	• CVLT [35] • VVM2 [36] subscales “construction 2” and “city map 2”	Short Delay Free Recall, Long Delay Free Recall, Short Delay Cued Recall, Long Delay Cued Recall, Learning Slope, List A Immediate Free Recall Trial 1–5, Learning Efficiency (List A Trial 5) Subtest “city map” (visuo-spatial memory), Subtest “construction” (verbal memory)
	Short-term memory	• CBTT (subtest of the WMS-R) forward task [61] • Digit Span test (subtest of the WMS-R) forward task [61] • List A Trial 1 of CVLT [35] • List B of CVLT [35] • VVM2 [36] subscales “construction 1” and “city map 1”	
	Working Memory	• CBTT backwards task [36, 61] • Digit Span test (subtest of the WMS-R) backwards task [61]	

*CBTT* Corsi Block Tapping Test, *CVLT* California Verbal Learning Test, *RT* reaction time, *TAP* Test of Attentional Performance, *VVM* Visual and Verbal Memory Test, *WMS* Wechsler Memory Scale. Parallel forms of the memory tests were used to avoid learning effects

### Description of both NF training protocols, EEG data recording and analysis

**Fig. 2:**
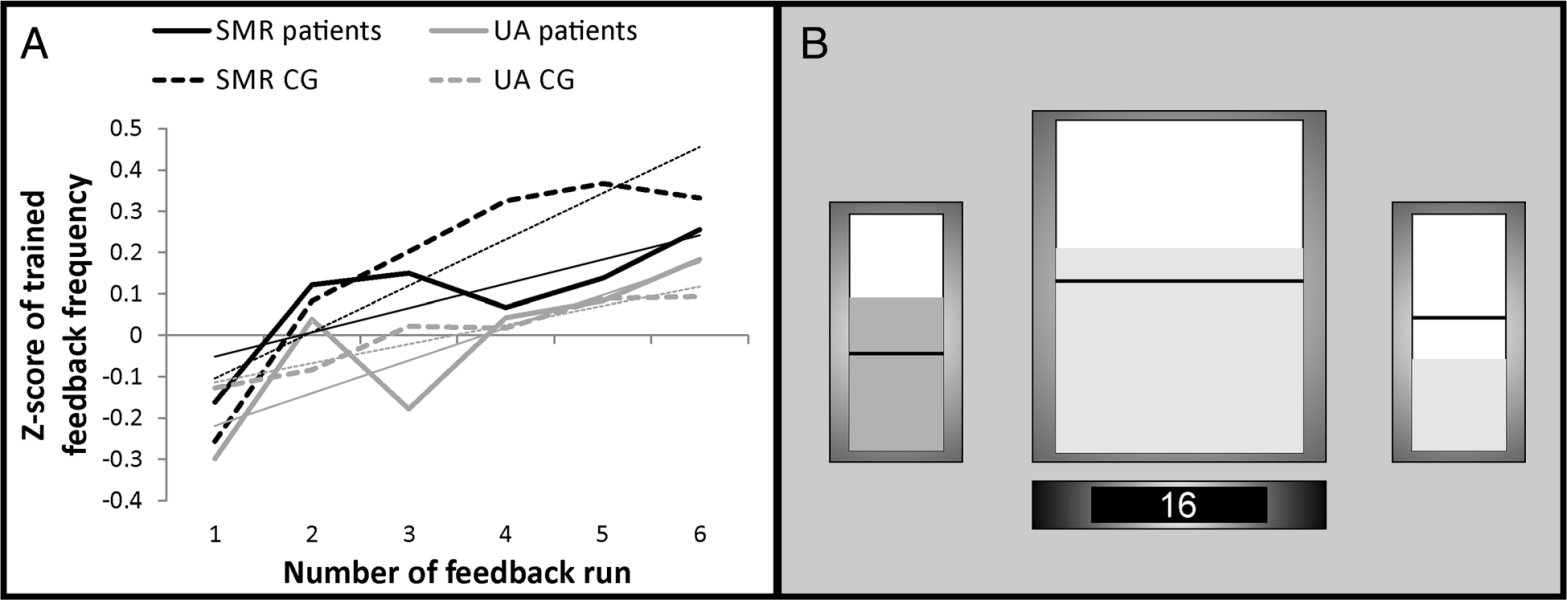
**a** Neurofeedback (NF) performance. Z-transformed EEG power for the two feedback frequency bands (SMR or UA) over the six NF training runs, presented separately for the two patient groups and two control groups. Values were averaged over all repeated NF training sessions. The regression lines for each group are indicated by finer lines. **b** Feedback screen. The amplitude of the relevant feedback frequency (either SMR or UA) was represented by the bar in the middle of the screen. The two smaller bars on the left and on the right side of the screen represented the inhibit bands (eye blinks or eye movements, muscle activity). The horizontal lines represented the individually defined thresholds for each bar (for details see methods section). The counter at the bottom indicated the number of reward points accumulated during the feedback runs: it increased whenever the middle bar was above and the left and right bars were below their individually defined thresholds

For both NF training protocols, EEG signal was recorded using a 10-channel amplifier (NeXus-10 MKII, Mind Media BV) with a sampling frequency of 256 Hz; the ground was located at the right mastoid, the reference was placed at the left mastoid. Furthermore, one EOG channel was recorded at the left eye. The NF paradigms were generated by using the software BioTrace + (Mind Media BV, [38]). Up to ten NF training sessions were carried out on different days three to five times per week. Each session lasted approximately 45 minutes and consisted of seven runs á three minutes each. The first run was a baseline run, in which participants were instructed to relax themselves and received no reward. The subsequent six runs were feedback runs, in which participants were instructed to try to modulate their brain activity in the desired direction. Participants received combined audio-visual feedback about their own brain activity. The feedback display contained three moving bars: One big bar in the middle and two smaller bars on the left and right side of the feedback screen (see Fig. 2b). During each three-minute feedback run the bars were continuously moving in a vertical direction. The height of the bar in the middle of the screen reflected the absolute power of the trained EEG frequency (12–15 Hz for SMR NF protocol, 2 Hz above the individually defined Alpha peak for the Upper Alpha NF protocol) in real time. Whenever the band power reached an individually predefined threshold (mean power value during the baseline run and the previous feedback runs) during the feedback runs, the color of this bar changed from red to green and participants were rewarded by getting points, which were also displayed at the feedback screen (reward counter). Furthermore, auditory feedback was provided as a reward by means of a midi tone feedback. When the bar in the middle of the screen was below the threshold it turned red again, the reward counter stopped and no tone was presented. Participants were instructed to try to voluntarily increase this bar. The threshold for the middle bar was adapted after each run. In order to prevent augmentation of the trained EEG frequency by artifacts, such as movements or eye blinks, two inhibit-bands were used, represented on the screen by the two smaller vertical moving bars on the left and right side of the display. The small bar on the left side of the feedback screen indicated eye blinks or eye movements. The height of the left bar reflected the absolute power between 0.05-10 Hz of the EOG channel. The small bar on the right side of the screen depicted muscle activity. The height of the right bar reflected the absolute power between 75–100 Hz of the feedback electrode [39, 40]. Artifact rejection thresholds were set for each participant individually (mean of baseline run + 1 SD), suspending feedback when eye-movements or other muscle activity caused gross EEG fluctuations. Hence, participants were instructed to keep these two bars as small as possible, but they were not told that they could influence the height of these bars by muscle activity or eye-movements. Participants were not rewarded when these two controlling bars were above their respective thresholds even when the trained EEG frequency was above its individually defined threshold.

For the SMR NF protocol, participants had to increase their SMR (12–15 Hz) activity recorded over Cz (according to the international 10–20 EEG placement system), since SMR is generally most pronounced over central scalp regions over the sensorimotor cortex [41]. For the Upper Alpha NF, we defined the individual Alpha frequency (IAF) of each single participant. Therefore, participants performed resting measurements with open and closed eyes á two minutes before the start of the NF training. These resting measurements were used to calculate the EEG power spectrum for each participant. EEG power spectra were calculated using Fast Fourier Transformation (FFT). FFT was computed for the segmented resting measurements (segment length 1 s) with maximum resolution of ~0.98 Hz. Furthermore, a 10 % Hanning window was applied including a variance correction to preserve overall power. Afterwards, peak detection in the Alpha frequency range was performed to identify the IAF. The Upper and the Lower Alpha band were defined in the following way [25]:

$$ \begin{array}{l}\mathsf{Lower}\kern0.5em \mathsf{A}\mathsf{l}\mathsf{pha}=\left(\mathsf{l}\mathsf{A}\mathsf{F}\hbox{-} \mathsf{2}\kern0.5em \mathsf{Hz}\right)\kern0.5em \mathsf{t}\mathsf{o}\kern0.5em \mathsf{l}\mathsf{A}\mathsf{F}\\ {}\mathsf{Upper}\kern0.5em \mathsf{A}\mathsf{l}\mathsf{pha}=\mathsf{l}\mathsf{A}\mathsf{F}\kern0.5em \mathsf{t}\mathsf{o}\kern0.5em \left(\mathsf{l}\mathsf{A}\mathsf{F}+\mathsf{2}\kern0.5em \mathsf{Hz}\right)\end{array} $$

The individually defined Upper Alpha frequency was used as feedback frequency for the Upper Alpha NF protocol. Participants should try to increase the Upper Alpha power recorded over Pz during NF training, since alpha oscillations are generally most pronounced over parieto-occipital areas [25].

Data analysis of EEG recordings was performed offline using the Brain Vision Analyzer software (version 2.01, Brain Products GmbH, Munich, Germany). Artifacts (e.g. eye blinks/movements, muscle activity) were rejected by means of a semi-automatic artifact rejection (criteria for rejection: > 50.00 μV voltage step per sampling point, absolute voltage value > ±100.00 μV). To analyze the feedback training data, absolute values of SMR (12–15 Hz) and Upper Alpha (IAF to (IAF + 2 Hz)) power were calculated and averaged separately for each three-minute run of each session using the Brain Vision Analyzer’s built-in method of complex demodulation. The complex demodulation is based on the complex (analytical) signal of a time series, where all frequencies except the one of interest are filtered out [42, 43].

### Description of statistical analysis

In order to analyze the NF performance, we determined the time course of the trained feedback frequency (either SMR power or Upper Alpha power) averaged over the ten NF training sessions across the six feedback runs. Therefore, regression analyses were carried out separately for each group (predictor variable = feedback run number; dependent variable = z-transformed power of the feedback frequency). In addition, one-sample t-tests were calculated for each group to verify the consistency of the learning effects. For statistical analyses and better comparability of the data between groups, SMR and Upper Alpha power values were z-transformed. The probability of a Type I error was maintained at 0.05. Holm corrections for multiple comparisons were applied [44].

For statistical analysis, T-scores of the single neuropsychological test parameters were used. To investigate the effects of NF on cognitive performance, we conducted intra-individual comparisons between cognitive performance assessed during pre- and post-assessment by using critical difference analysis [45, 46]. To establish the critical difference for a pair of test scores, a correction for measurement error based on the test-retest reliability of the test is performed. The test-retest reliability is defined as the variation in measurements taken by a single subject or instrument on the same task, under the same conditions, and in a short period of time. It describes the consistency and stability of a measure over time [47]. To identify significant improvement or decline for each participant, the critical difference of the relevant test parameter was compared with the test score difference obtained during the post-assessment minus the pre-assessment. A test parameter is considered significant when the difference between pre- and post-assessment shown by the single participants is larger than the critical difference, which can be detected by each test and only occurs in the population with a probability lower than α < 10 %. When the test-retest reliability for a given psychological test is low (e.g. <.60), even large test score differences between pre- and post-test cannot be distinguished from random noise. In contrast, when the test-retest reliability is very high (e.g. >.90), every difference between pre- and post-test will be highly significant though in many cases of no clinical relevance. The test-retest reliability of the tests used in the present investigation lay in a moderate to high range. Differences in T-scores between pre- and post-assessment for each cognitive test parameter were compared with critical differences on the single subject level as well as on the group level. Furthermore, we calculated the probability that the number of significant performance improvements and declines were observed by chance alone using the binomial model. Given measurement independency across participants and the probability of one single participant reaching the critical difference of *p* = 0.01, each statistical comparison evaluated the proportion of successes (performance differences between post- and pre-assessment > critical differences) in relation to the total number of comparisons. These probability values were corrected for multiple comparisons using false discovery rates [48].

## Results

### NF performance

Stroke patients as well as healthy controls were able to voluntarily modulate brain rhythms during NF training (Fig. 2a). This was reflected in a linear increase of power in the target frequency band. For the SMR patient group, regression analysis revealed linear changes of z-transformed SMR power over the six training runs within the NF training sessions (*F*(1,5) = 6.37, *p* = 0.05). The regression model explained 61 % of variance of SMR power over the training runs. Eight out of eleven patients (73 %) receiving SMR NF training were able to linearly increase their SMR power over the training runs. One sample t-tests revealed that the individual regression slopes of the SMR patient group differed significantly from zero (*t*(10) = 2.38, *p* < 0.05). For the Upper Alpha patient group, the regression model was significant (*F*(1,5) = 8.25, *p* < 0.05) and explained 67 % of variance of Upper Alpha power over the training runs. Four out of six (67 %) patients of the Upper Alpha group showed a positive slope, indicating that they were able to linearly increase their individually defined Upper Alpha power over the feedback runs within the NF training sessions. One sample t-tests revealed that the individual regression slopes of the Upper Alpha patient group differed by trend significantly from zero (*t*(5) = 2.15, *p* = 0.08). In sum, 12 out of 17 patients (70 %) were able to linearly increase their EEG power. The SMR CG also showed a significant linear increase in SMR power over the NF training runs (*F*(1,5) = 14.58, *p* < 0.05). The regression model explained 78 % of variance of SMR power over the training runs and 11 out of 16 participants (69 %) showed a positive slope (*t*(15) = 2.08, *p* = 0.05). For the UA control group the regression model was also significant (*F*(1,5) = 45.73, *p* < 0.01) and explained 92 % of variance of Upper Alpha power over the training runs. Nineteen out of 24 participants (79 %) of the UA CG group showed a positive slope over the runs (*t*(23) = 1.80, *p* = 0.08). A repeated measures ANOVA with the between subject factor group revealed no differences in the regression slopes between groups (*F*(1,3) = 0.64, *ns.*). There were no significant changes in SMR or UA power across the feedback training sessions neither in the patient nor in the control groups.

### Cognitive performance – comparison between pre- and post-assessment – group level

**Fig. 3:**
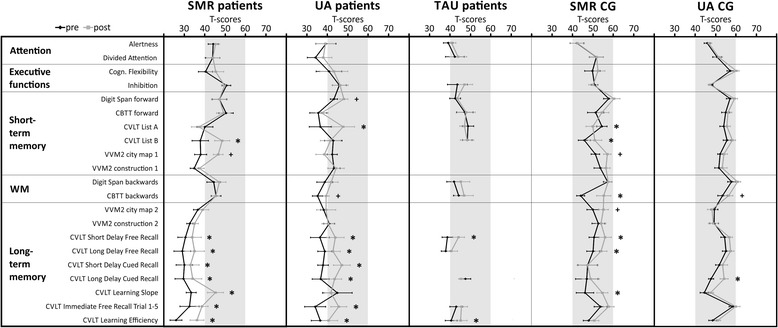
Test performance is expressed in T-scores with population mean *M* = 50 and standard deviation *SD* = 10. Group average test scores and standard errors for measurements of attention, executive functions, short- and long-term memory, and working memory (WM) performed during the pre- and post-assessment are depicted separately for stroke patients and healthy controls. Significant differences between pre- and post-test (critical difference analysis on the group level, [45, 46]) are marked with asterisks (*significant, ^+^marginally significant). CBTT, Corsi Block Tapping Test; CVLT, California Verbal Learning Test; VVM, Visual and Verbal Memory Test

After NF training, the SMR patient group showed significant performance improvements in parameters of the CVLT assessing verbal short- (“List B”) and long-term memory (all CVLT test parameters) compared to the pre-assessment (Fig. 3). Furthermore, SMR patients showed a numerical performance improvement in visual-spatial short-term memory (VVM2 subscale “city map 1”), which slightly failed to reach the significance level. Comparable to the results of the SMR patient group, the UA patient group showed significant performance improvements in the CVLT parameters assessing verbal long-term memory, except for the test parameter “Learning slope” (Fig. 3). UA patients also improved their verbal short-term memory performance (CVLT “List A”) as well as their memory span (Digit Span forwards task), which was significant by trend. Additionally, the UA patients showed a numerical improvement in the CBTT backwards task assessing WM, although the difference between pre- and post-test T-scores was marginally lower than the critical difference. The TAU patient group showed the least cognitive improvements of all patient groups after cognitive training compared to the pre-assessment. Performance improvements could be observed in two parameters of the long-term memory tasks: “short delay free recall” and “learning efficiency”.

Healthy controls showed cognitive improvements due to NF training as well, which were comparable to cognitive improvements of stroke patients. Comparable to the results of the SMR patient group, the SMR CG also showed significant performance improvements in verbal short- (CVLT List B) and long-term memory (CVLT short and long delay free recall and learning slope) as well as in visual-spatial short- (VVM2 city map 1) and long-term memory (VVM2 city map 2) when comparing the post- and pre-assessment (Fig. 3). Furthermore, the SMR CG improved in WM performance (CBTT backwards task). This is the only NF training group that also showed decreases in cognitive performance on the group level when comparing pre- and post-assessment. After the NF training, the SMR CG showed a lower performance in the CVLT parameter “List A” assessing short-term memory performance compared to the pre-test, although the T-score of 49.81 reached by the SMR CG during the post-assessment is still in the normal range. The Upper Alpha CG showed the fewest significant performance improvements when comparing the pre- and post-assessment (Fig. 3). Note that this CG already showed the highest cognitive performance during the pre-assessment compared to the other groups. After NF training, the Upper Alpha CG significantly improved its performance in the CVLT parameter “long delay cued recall” assessing long-term memory performance compared to the pre-test. As the UA patient group, the UA CG group showed a numerical improvement in the CBTT backwards task assessing WM when comparing pre- and post-assessment, although this difference between pre- and post-test T-scores was marginally lower than the critical difference.

### Cognitive performance – comparison between pre- and post-assessment – single subject level

**Fig. 4:**
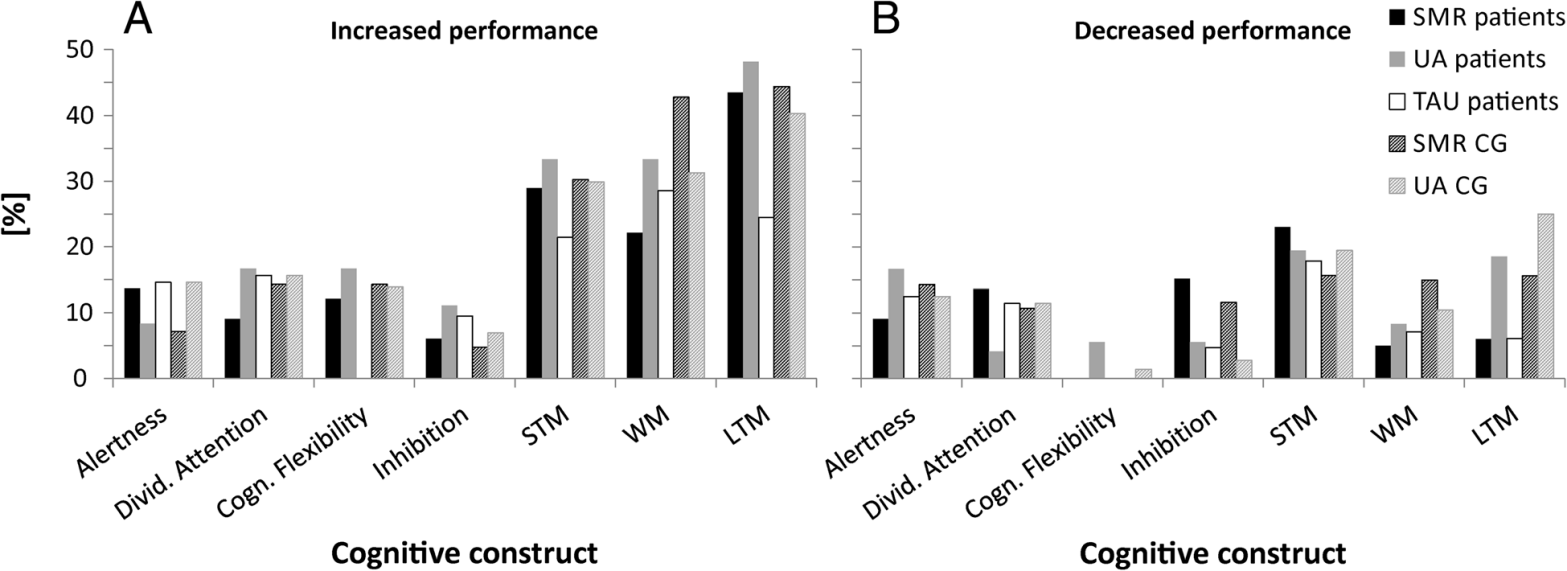
Individual improvements and declines in cognitive performance after training, presented separately for stroke patients and healthy controls. Percentage of participants per group showing either increased (**a**) or decreased (**b**) performance in the different cognitive constructs (short-term STM, long-term LTM, and working memory WM) when comparing the pre- and post-assessment

For each group, we determined the number of participants showing significantly increased, constant or decreased cognitive performance by counting the number of pre-post differences scores larger than the test specific critical differences and dividing this amount by the number of measurements per construct and participants [45, 46]. All groups showed the strongest performance improvements in memory tests (Fig. 4). Cognitive decline was present after training but never markedly over 20 % and therefore attributable to random performance fluctuations and not to any deleterious NF training effects (Fig. 4b) [45, 46]. Importantly, cognitive decline was comparable across groups. Statistical comparisons using chi-square tests revealed that the number of participants showing increased, constant or decreased cognitive performance was comparable between groups (all *p*-values > 0.10). SMR and UA NF training led to comparable individual improvements and decline in cognitive performance. The TAU group showed the lowest percentage of cognitive improvement in short- and long-term memory tasks compared to the NF training groups.

The probability that the numbers of significant performance improvements and declines were observed by chance alone is depicted in Table 3. After correction for multiple comparisons using false discovery rates [48], substantial improvements in measurements of short-term memory and long-term memory could be detected among patients and controls who performed NF training. Healthy controls also showed significant improvements in working memory. The probability that the observed performance improvements of the NF training groups were attributable to random noise alone was rather low, as indicated by the p-values shown in Table 3. The TAU group showed no significant improvements any more. No significant decrease in performance could be observed in any of the patient groups. The UA CG showed significant performance declines in short- and long-term memory tasks. Here, we would like to note that four participants of the UA CG were responsible for this effect. Theses participants showed performance declines in most of the cognitive constructs, regardless of their functional connection with UA rhythm. In our view these results are more easily explained in terms of a general decrease in motivation in these four participants, rather than representing genuine deleterious effects of UA NF.

**Table 3:** Probability that the number of successes (performance differences between post- and pre-assessment > critical differences) is due to chance alone given a probability of success for each individual comparison of *p* = 0.01 and the total number of comparisons performed in each group.

(A) Probability that chance alone is responsible for cognitive improvements after training and total number of comparisons performed in each group							
	Alertness	Divided Attention	Cogn. Flexibility	Inhibition	WM	STM	LTM
SMR patients	0.38 (22)	0.65 (44)	0.42 (33)	0.86 (33)	0.17 (22)	1.64 × 10^−5^ (66)*	2.66 × 10^−15^ (99)*
UA patients	0.72 (12)	0.21 (24)	0.27 (18)	0.55 (18)	0.03 (12)	1.25 × 10^−4^ (36)*	1.11 × 10^−12^ (54)*
TAU patients	0.42 (14)	0.95 (28)		0.64 (21)	0.04 (14)	0.06 (28)	0.02 (35)
SMR CG	0.97 (32)	0.89 (64)	0.87 (48)	0.99 (48)	0.003 (32)*	3.50 × 10^−8^ (96)*	3.55 × 10^−15^ (144)*
UA CG	0.20 (48)	0.05 (96)	0.18 (72)	0.86 (72)	4.35 × 10^−5^ (48)*	3.12 × 10^−11^ (144)*	<1 × 10^−35^ (216)*
(B) Probability that chance alone is responsible for cognitive declines after training and total number of comparisons performed in each group							
SMR patients	0.66 (22)	0.27 (44)	1.00 (33)	0.23 (33)	0.90 (22)	0.002 (66)	0.94 (99)
UA patients	0.34 (12)	0.92 (24)	0.85 (18)	0.85 (18)	0.72 (12)	0.06 (36)	0.04 (54)
TAU patients	0.77 (14)	0.79 (28)		0.89 (21)	0.77 (14)	0.14 (28)	0.69 (35)
SMR CG	0.84 (32)	0.96 (64)	1.00 (48)	0.87 (48)	0.40 (32)	0.05 (96)	0.37 (144)
UA CG	0.35 (48)	0.36 (96)	0.99 (72)	0.99 (72)	0.53 (48)	0.0005 (144)*	<1 × 10^−35^ (216)*

Probability that chance alone is responsible for improvements (A) and declines (B) in cognitive performance after training, presented separately for stroke patients and healthy controls for the different cognitive constructs (short-term STM, long-term LTM, and working memory WM). Uncorrected p-values, which were significant after correction for multiple comparisons employing false discovery rates [48], are marked with asterisks.

## Discussion

The aim of the present study was twofold. First, we investigated whether stroke patients were able to learn to modulate their own EEG activity by means of NF training. Second, we evaluated the effects of two different NF training protocols on cognition, especially memory, in stroke patients to demonstrate its feasibility and usefulness as cognitive rehabilitation tool.

In a first step, we could show that stroke patients were able to voluntarily increase their EEG activity within the NF training sessions in the trained frequency range (either SMR or UA). NF performance of stroke patients and healthy controls was comparable. All groups showed a linear increase in the trained frequency band over the feedback runs, indicating successful NF training [9]. In line with previous findings in healthy participants, about 30 % of patients were not able to modulate their EEG activity [49]. The inability to control the own brain activity may be attributed to different factors such as differences in brain structure [49], inter-individual differences in neurophysiological and psychological factors, or cognitive strategies [38, 50, 51]. Furthermore, the type and localization of brain lesion might explain that part of stroke patients were not able to modulate their brain activity. However, there was no clear relationship between lesion location and the ability to up-regulate SMR or UA power in our study (see Table 1). For instance, some patients were able to increase the feedback frequencies (e.g. patient code 6 and 16), while others were not (e.g. patient code 10 and 15), although they showed comparable lesion locations (e.g. arteria cerebri media). Prior neuroimaging studies revealed a network of different brain regions, which seemed to be involved in more general cognitive regulatory mechanisms that are engaged during NF. This cognitive control network included the insula, anterior cingulate cortex, supplementary motor and dorsomedial and lateral prefrontal areas [52, 53]. Based on the present vague description of lesion locations, it is difficult to determine whether stroke patients of the present study had lesions in this network or not. For instance, a lesion in the arteria cerebri media can affect many different brain areas. Nevertheless, patients of the present study showed no lesions in the arteria cerebri anterior. This implies that the anterior cingulate cortex should have been intact, which is part of this cognitive control network involved in NF [52, 53]. Another study by Birbaumer et al. (2013) postulated that the basal ganglia would play a critical role in NF [54]. However, we could not support this assumption since we had two patients with lesions in the basal ganglia, one was able to increase SMR power (patient code 11) while the other was not (patient code 3). Future studies with an exact determination of the brain lesion are necessary to draw a meaningful conclusion concerning the relationship between lesion location and ability to control EEG rhythms by means of NF training.

We did not find any consistent changes in SMR or UA power across the NF training sessions. This is in line with prior studies in healthy people [9, 12, 55, 56], which also found robust modulations of EEG power values within sessions but not across sessions. Hence, our results provide evidence that stroke patients and healthy controls were able to adaptively modulate SMR or UA activity according to task demands at a given time (within a NF training session), which does not necessarily imply that the average SMR/UA baseline level of a feedback user has to change [9]. The ability to upregulate SMR/UA power voluntarily at a given time might also explain the improvements in cognitive performance after NF training compared to the pre-test. In a recent study, Kober et al. (2015) also found significant changes in SMR power within NF training sessions but not across NF training sessions [9]. After NF training, healthy participants showed significant correlations between SMR power and event-related potentials (P300, N1) in the EEG, which are generally indicators for stimulus processing intensity, during the performance of a memory task. A higher SMR power during the memory task was associated with a more intense stimulus processing and consequently with an improved memory performance after NF training compared to the pre-test [9]. Hence, although no changes in absolute SMR power across NF training sessions could be observed, these prior findings indicate that participants can learn to voluntarily increase SMR power at a given time, for instance during performing a memory test, which goes along with improved cognitive performance. In the present study, it was not possible to perform multi-channel EEG measurements in stroke patients at the clinic during the pre- and post-assessment. Future studies are needed to investigate whether stroke patients are also able to transfer the mental state reached during successful NF training to other situations without real-time feedback of own brain activity.

In a second step, we investigated the NF training outcome. Independent of the used NF protocol, stroke patients and healthy controls showed more general improvements in verbal short- and long-term memory performance after NF training supporting prior findings in healthy people [7–12, 18–24] and post-stroke victims [28–31, 33]. An explanation for these comparable effects of SMR and UA NF on verbal memory may be that both EEG frequencies are associated with improved stimulus processing, which should foster the storage and retrieval of information and might explain the overlapping effects of SMR and UA NF [9, 15, 26]. Furthermore, the SMR and UA frequency range may overlap in some cases. Participants who received SMR NF and at the same time showed a high individual Alpha frequency (e.g. 12 Hz, resulting in an UA frequency range of 12–14 Hz, [25]) might be actually modifying their power in the UA range. Considering the importance of UA for cognitive performance, one might assume that at least part of the effects found for SMR based NF training might be due to the influence of UA activity [57].

Specific effects of SMR and UA NF training on memory functions were observed as well. The SMR patient group showed additional and specific improvements in visuo-spatial short-term memory performance. Prior SMR based NF studies also found performance improvements in visual-spatial tasks, such as mental rotation tasks [40]. However, the majority of prior SMR NF studies reported on specific improvements in verbal memory tasks in young adults [8–12]. In contrast, our results in older stroke patients and older healthy controls indicate that SMR NF seems to have specific positive effects on visuo-spatial memory, including short- and long-term memory as well as WM (improvement in CBTT backwards task in the SMR CG). Furthermore, SMR patients showed the strongest performance improvements in their learning efficiency, which is a measure of memory consolidation, probably due to a more intense stimulus processing after SMR based NF [9, 15, 35].

The UA patient group showed additional and specific improvements in WM performance. Prior NF studies that found positive effects of NF training on WM performance also trained EEG frequencies in the Alpha range such as the total Alpha band (about 8–12 Hz), Upper Alpha (about 10–12 Hz), or the Alpha peak frequency [22, 23]. Furthermore, in line with previous findings in healthy people, the UA patient group showed strong performance improvements in verbal short-term memory and memory capacity after NF training [12, 18, 20, 21, 23].

NF training had no effects on other cognitive functions. The majority of prior NF studies also linked SMR and UA to memory functions, whereas performance improvements in other cognitive functions such as attention, inhibitory control, or cognitive flexibility were mainly found in T/B based NF training studies [7, 8, 33, 58, 59]. In the NF literature, there are issues concerning specificity of NF training [8]. Our results indicate that there is specificity and independence regarding cognitive outcome such that performance enhancement in memory functions is specific to changes in the SMR and UA frequency band while leaving other cognitive functions unchanged.

NF training was more efficient than traditional cognitive rehabilitation. The TAU group also showed improvements in long-term memory functions after traditional cognitive training compared to the pre-assessment, which supports prior findings [5]. However, these memory improvements were not as strong as in the NF patient groups. After correction for multiple comparisons, the TAU group showed no significant performance improvements any more. Based on these results, one may exclude that the NF training effects can be merely attributed to placebo-effects or spontaneous cognitive improvements. NF training might be more efficient than traditional cognitive training because with NF training one can directly modulate brain activation patterns that underlie cognitive functions. This underlines the potential usefulness of NF training as cognitive rehabilitation tool.

After NF training, all groups showed a small decrease in cognitive performance compared to the pre-test. However, cognitive decline due to NF training was insignificant and did not differ between stroke patients and healthy controls. Therefore, one may conclude that SMR and UA NF do not have deleterious effects on the electrophysiological balance or associated cognitive functions [2]. Reasons for this cognitive decline after NF training, e.g. changes in motivation, mood or fatigue, have to be investigated in more detail in future studies. In the present study, all stroke patients showed more or less circumscribed memory deficits, but no severe deficits in further cognitive functions. Hence, we could demonstrate that NF is a tool to treat moderate memory deficits in post-stroke victims.

The patient inclusion and exclusion criteria used in the present study determine the limits of generalization of the present findings to other patient populations. For instance, the effects of NF on stroke patients with aphasia have to be clarified in future studies. All stroke patients showed memory deficits, but no severe deficits in other cognitive functions. As our patient population differed in brain lesion sites, we cannot exclude potential side-effects of the individual lesions on learning. This issue clearly calls for future studies that incorporate detailed anatomical data. Further limitations of the present study are the small sample size and the heterogeneity of patients concerning post-stroke delays and cause of brain lesion. Additionally, no follow-up measurements were performed. Hence, we cannot draw any conclusion concerning long-term effects of NF training at this stage of research.

## Conclusions

In this proof-of-principle study, we could show that post-stroke victims were as successful in modulating their own brain activity by means of NF training as healthy controls. Furthermore, SMR and UA based NF training had specific positive effects on memory functions in stroke patients and healthy controls. NF training effects were even stronger than effects of traditional cognitive rehabilitation methods in stroke patients. Hence, compared to prior single-case studies in stroke patients [28–32], we could (i) demonstrate the specificity of different NF training protocols in a larger sample of stroke patients, (ii) compared NF related training effects in stroke patients to the effects of treatment as usual in stroke survivors, (iii) used a comprehensive neuropsychological test battery to assess possible effects of NF on many different cognitive functions, and (iv) reported on possible negative effects of NF training for the first time. The NF training protocols were feasible for stroke patients with memory deficits and may represent a new rehabilitation strategy suitable to overcome some of the usual pitfalls of traditional cognitive rehabilitation. Hence, NF seems to be an alternative, innovative and easy-to-use cognitive rehabilitation tool since the electrical activity of the brain is affected directly and, therefore, the cortical substrates of cognitive functions themselves [6].

## Abbreviations

Bt: bilateral
CBTT: corsi block tapping test
CG: Control group
CVLT: California Verbal Learning Test
EEG: electroencephalogram
F: female
FFT: fast fourier transformation
IAF: individual alpha frequency
Lt: left
LTM: long-term memory
M: male
MMSE: mini-mental state examination
NF: neurofeedback
RT: reaction time
Rt: right
SD: standard deviation
SE: standard error
SMR: sensorimotor rhythm
STM: short-term memory
TAP: test of attentional performance
TAU: treatment as usual
T/B: Theta/Beta
TBI: traumatic brain injury
UA: upper alpha
VVM: visual and verbal memory test
WM: working memory
WMS: Wechsler Memory Scale

## References

[1] Melkas S, Jokinen H, Hietanen M, Erkinjuntti T (2014). Poststroke cognitive impairment and dementia: prevalence, diagnosis, and treatment. DNND.

[2] Thornton KE, Carmody DP (2009). Traumatic Brain Injury Rehabilitation: QEEG Biofeedback Treatment Protocols. Appl Psychophysiol Biofeedback.

[3] Hoffmann T, Bennett S, Koh C, McKenna K (2010). A systematic review of cognitive interventions to improve functional ability in people who have cognitive impairment following stroke. Top Stroke Rehabil.

[4] Langhorne P, Bernhardt J, Kwakkel G (2011). Stroke rehabilitation. Lancet.

[5] Elliott M, Parente F (2014). Efficacy of memory rehabilitation therapy: A meta-analysis of TBI and stroke cognitive rehabilitation literature. Brain Inj.

[6] Nelson L (2007). The Role of Biofeedback in Stroke Rehabilitation: Past and Future Directions. Top Stroke Rehabil.

[7] Kropotov JD (2009). Quantitative EEG, event-related potentials and neurotherapy.

[8] Gruzelier JH (2014). EEG-neurofeedback for optimising performance. I: A review of cognitive and affective outcome in healthy participants. Neurosci Biobehav Rev.

[9] Kober SE, Witte M, Stangl M, Valjamae A, Neuper C, Wood G (2015). Shutting down sensorimotor interference unblocks the networks for stimulus processing: An SMR neurofeedback training study. Clin Neurophysiol.

[10] Hoedlmoser K, Pecherstorfer T, Gruber G, Anderer P, Doppelmayr M, Klimesch W (2008). Instrumental conditioning of human sensorimotor rhythm (12–15 Hz) and its impact on sleep as well as declarative learning. Sleep.

[11] Schabus M, Heib DP, Lechinger J, Griessenberger H, Klimesch W, Pawlizki A (2014). Enhancing sleep quality and memory in insomnia using instrumental sensorimotor rhythm conditioning. Biol Psychol.

[12] Vernon D, Egner T, Cooper N, Compton T, Neilands C, Sheri A (2003). The effect of training distinct neurofeedback protocols on aspects of cognitive performance. Int J Psychophysiol.

[13] Ullman MT (2004). Contributions of memory circuits to language: the declarative/procedural model. Cognition.

[14] Pfurtscheller G (1981). Central beta rhythm during sensorimotor activities in man. Electroencephalogr Clin Neurophysiol.

[15] Sterman MB (1996). Physiological origins and functional correlates of EEG rhythmic activities: implications for self-regulation. Biofeedback Self Regul.

[16] Sterman MB (2000). Basic concepts and clinical findings in the treatment of seizure disorders with EEG operant conditioning. Clinical EEG Electroencephalography.

[17] Pfurtscheller G (1992). Event-related synchronization (ERS): an electrophysiological correlate of cortical areas at rest. Electroencephalogr Clin Neurophysiol.

[18] Escolano C, Aguilar Herrero M, Minguez J (2011). EEG-Based Upper Alpha Neurofeedback Training Improves Working Memory Performance. 33rd Annual International IEEE EMBS Conference, August 30 - September 3, 2011.

[19] Escolano C, Olivan B, Lopez-del-Hoyo Y, Garcia-Campayo J, Minguez J (2012). Double-blind single-session neurofeedback training in upper-alpha for cognitive enhancement of healthy subjects. Conf Proc IEEE Eng Med Biol Soc.

[20] Escolano C, Navarro-Gil M, Garcia-Campayo J, Minguez J (2013). EEG-based upper-alpha neurofeedback for cognitive enhancement in major depressive disorder: A preliminary, uncontrolled study. Conf Proc IEEE Eng Med Biol Soc.

[21] Escolano C, Navarro-Gil M, Garcia-Campayo J, Congedo M, De_Ridder D, Minguez J (2014). A controlled study on the cognitive effect of alpha neurofeedback training in patients with major depressive disorder. Front Behav Neurosci.

[22] Angelakis E, Stathopoulou S, Frymiare JL, Green DL, Lubar JF, Kounios J (2007). EEG Neurofeedback: A Brief Overview and an Example of Peak Alpha Frequency Training for Cognitive Enhancement in the Elderly. Clin Neuropsychol.

[23] Nan W, Rodrigues JP, Ma J, Qu X, Wan F, Mak P (2012). Individual alpha neurofeedback training effect on short term memory. Int J Psychophysiol.

[24] Michels L, Moazami-Goudarzi M, Jeanmonod D, Sarnthein J (2008). EEG alpha distinguishes between cuneal and precuneal activation in working memory. Neuroimage.

[25] Klimesch W (1999). EEG alpha and theta oscillations reflect cognitive and memory performance: a review and analysis. Brain Res Brain Res Rev.

[26] Klimesch W, Sauseng P, Hanslmayr S (2007). EEG alpha oscillations: The inhibition–timing hypothesis. Brain Res Rev.

[27] May G, Benson R, Balo R, Boutros N (2013). Neurofeedback and traumatic brain injury: A literature review. Ann Clin Psychiatry.

[28] Rozelle GR, Budzynski TH (1995). Neurotherapy for stroke rehabilitation: a single case study. Biofeedback Self Regul.

[29] Bearden T, Cassisi J, Pineda M (2003). Neurofeedback Training for a Patient with Thalamic and Cortical Infarctions. Appl Psychophysiol Biofeedback.

[30] Doppelmayr M, Nosko H, Pecherstorfer T, Fink A (2007). An Attempt to Increase Cognitive Performance After Stroke With Neurofeedback. Biofeedback Self Regul.

[31] Putman J (2002). EEG Biofeedback on a Female Stroke Patient with Depression: A Case Study. J of Neurotherapy.

[32] Cannon KB, Sherlin L, Lyle RR (2010). Neurofeedback Efficacy in the Treatment of a 43-Year-Old Female Stroke Victim: A Case Study. J Neurother.

[33] Hofer D, Kober SE, Reichert J, Krenn M, Farveleder K, Grieshofer P (2014). Spezifische Effekte von EEG basiertem Neurofeedbacktraining auf kognitive Leistungen nach einem Schlaganfall: Ein nutzvolles Werkzeug für die Rehabilitation?. Lernen und Lernstörungen.

[34] Kessler J, Markowitsch HJ, Denzler P (1990). Mini Mental Status Examination MMSE: German Version.

[35] Niemann H, Sturm W, Thöne-Otto A, Willmes K (2008). CVLT - California Verbal Learning Test.

[36] Schelling D, Schächtele B (2001). VVM. Visueller und Verbaler Merkfähigkeitstest.

[37] Bounias M, Laibow RE, Bonaly A, Stubblebine AN (2002). EEG-NeuroBioFeedback Treatment of Patients with Brain Injury: Part 1: Typological Classification of Clinical Syndromes. J Neurother.

[38] Kober SE, Witte M, Ninaus M, Neuper C, Wood G (2013). Learning to modulate one’s own brain activity: the effect of spontaneous mental strategies. Front Hum Neurosci.

[39] Weber E, Köberl A, Frank S, Doppelmayr M (2011). Predicting Successful Learning of SMR Neurofeedback in Healthy Participants: Methodological Considerations. Appl Psychophysiol Biofeedback.

[40] Doppelmayr M, Weber E (2011). Effects of SMR and Theta/Beta Neurofeedback on Reaction Times, Spatial Abilities, and Creativity. J Neurother.

[41] Sterman MB, Howe RC, Macdonald LR (1970). Facilitation of Spindle-Burst Sleep by Conditioning of Electroencephalographic Activity While Awake. Science.

[42] Brain Products GmbH. BrainVision Analyzer 2.0.1 User Manual, (3rd ed.). Munich, Germany: Brain Products GmbH;2009.

[43] Draganova R, Popivanov D (1999). Assessment of EEG frequency dynamics using complex demodulation. Physiol Res.

[44] Holm S (1979). A Simple Sequentially Rejective Multiple Test Procedure. Scand J Stat.

[45] Huber HP (1973). Psychometrische Einzelfalldiagnostik.

[46] Huber HP (1977). Single case analysis. Behavioral analysis and modifications.

[47] Bortz J (2005). Statistik für Human- und Sozialwissenschaftler.

[48] Benjamini Y, Yekutieli D. The control of the false discovery rate in multiple testing under dependency. Ann. Statist 2001;29(4):1165–88.

[49] Allison B, Neuper C, Tan D, Nijholt A (2010). Could Anyone Use a BCI?. Brain-Computer Interfaces: Human-Computer Interaction Series.

[50] Witte M, Kober SE, Ninaus M, Neuper C, Wood G (2013). Control beliefs can predict the ability to up-regulate sensorimotor rhythm during neurofeedback training. Front Hum Neurosci.

[51] Wood G, Kober SE, Witte M, Neuper C (2014). On the need to better specify the concept of “control” in brain-computer-interfaces/neurofeedback research. Front Syst Neurosci.

[52] Ninaus M, Kober S, Witte M, Koschutnig K, Stangl M, Neuper C (2013). Neural substrates of cognitive control under the belief of getting neurofeedback training. Front Hum Neurosci.

[53] Ninaus M, Kober S, Witte M, Koschutnig K, Neuper C, Wood G (2015). Brain volumetry and self-regulation of brain activity relevant for neurofeedback. Biol Psychol.

[54] Birbaumer N, Ruiz S, Sitaram R (2013). Learned regulation of brain metabolism. Trends Cogn Sci.

[55] Vernon DJ (2005). Can Neurofeedback Training Enhance Performance? An Evaluation of the Evidence with Implications for Future Research. Appl Psychophysiol Biofeedback.

[56] Dempster T, Vernon D (2009). Identifying Indices of Learning for Alpha Neurofeedback Training. Appl Psychophysiol Biofeedback.

[57] Hanslmayr S, Sauseng P, Doppelmayr M, Schabus M, Klimesch W (2005). Increasing Individual Upper Alpha Power by Neurofeedback Improves Cognitive Performance in Human Subjects. Appl Psychophysiol Biofeedback.

[58] Arns M, de Ridder S, Strehl U, Breteler M, Coenen T (2009). Efficacy of Neurofeedback treatment in ADHD: The effects on Inattention, Impulsivity and Hyperactivity: A meta-analysis. Clin EEG Neurosci.

[59] Fox DJ, Tharp DF, Fox LC (2005). Neurofeedback: An Alternative and Efficacious Treatment for Attention Deficit Hyperactivity Disorder. Appl Psychophysiol Biofeedback.

[60] Zimmermann P, Fimm B (2007). Testbatterie zur Aufmerksamkeitsprüfung (TAP).

[61] Härting C, Wechsler D (2000). Wechsler-Gedächtnistest revidierte Fassung: WMS-R: Manual; deutsche Adaptation der revidierten Fassung der Wechsler Memory Scale.

